# 849. Intraoperative Phenylephrine Use Is Not Associated with Acute Kidney Injury in Patients Undergoing Burn Surgery

**DOI:** 10.1093/jbcr/irag033.319

**Published:** 2026-04-06

**Authors:** Samuel Boes, Sadaf Akbari, Colette Galet, Alexander Kurjatko

**Affiliations:** University of Iowa Carver College of Medicine, Iowa City, IA; University of Iowa Burn Center, Iowa City, IA; University of Iowa, Iowa City, IA; University of Iowa, Iowa City, IA

## Abstract

**Introduction:**

Phenylephrine is a vasopressor commonly used to manage intraoperative hypotension. A prior retrospective study demonstrated an independent association between intraoperative phenylephrine exposure and acute kidney injury (AKI) in adults undergoing noncardiac surgery. However, the association between AKI and intraoperative phenylephrine exposure has not been explored in burn-injured populations. Herein, we examined the association between intraoperative phenylephrine exposure and AKI and mortality among burn patients.

**Methods:**

This was a retrospective cohort study. We included all adult burn patients admitted to our institution’s burn treatment center from July 1, 2015 to June 30, 2024 who required at least burn-related one operation and had a postoperative serum creatinine value within 7 days of their first surgery. Patients admitted for other traumatic injuries (e.g., frostbite) were excluded. The primary exposure was intraoperative phenylephrine during the first burn-related surgery. Serum creatinine values from hospitalizations unrelated to the burn obtained within 90 days prior to burn presentation to our burn center were recorded as baseline. Primary outcomes were postoperative AKI and in-hospital mortality. Postoperative AKI was defined in accordance with Kidney Disease: Improving Global Outcomes (KDIGO) guidelines. Secondary outcomes were hospital length of stay and discharge disposition. All analyses were performed using SPSS 28.0 (IBM, Chicago, IL), and *p*<.05 was considered significant.

**Results:**

A total of 639 patients were included in this study; 393 received intraoperative phenylephrine. Patients in the phenylephrine group were significantly older (58 [42, 69] vs. 47 [33, 59] years, *p*<.001). There was no significant difference in postoperative AKI between the two groups (9.4% vs. 7.3%, *p*=.358) or in-hospital mortality (3.1% vs. 2.4%, *p*=.648). The phenylephrine group had longer hospital stays (14 [9, 22] vs. 11 [7, 17] days, *p*<.001), received more burn related surgeries (1 [1, 2] vs. 1 [1, 2], *p*=.017), was more likely to have preoperative AKI (27.8% vs. 18.3%, *p*=.007), and experienced postoperative hypotension more often (20.7% vs. 7.7%, *p*<.001) than the control group. Patients who received intraoperative phenylephrine were more likely to require higher level of care following discharge than the control group (*p*=.025).

**Conclusions:**

Intraoperative phenylephrine use was not associated with increased risk of postoperative AKI or mortality in our burn cohort but was linked to postoperative hypotension and longer hospitalization.

**Applicability of Research to Practice:**

The use of intraoperative phenylephrine is not associated with AKI in burn-injured patients. Further studies are warranted to evaluate the overall safety profile of phenylephrine in burn-injured patients.

**Funding for the study:**

N/A.

